# Effect of social app-assisted education and support on glucose control in patients with coronary heart disease and diabetes mellitus

**DOI:** 10.3389/fcvm.2022.947130

**Published:** 2022-09-23

**Authors:** Jing Zhong, Huimin Zhang, Zhuyu Li, Dehui Qian, Yingqian Zhang, Chao Li, Yuanbin Song, Zhexue Qin, Jie Yu, Shi-zhu Bian, Yang Yu, Ke Wang, Jing-Wei Li

**Affiliations:** ^1^Department of Cardiology, Xinqiao Hospital, Army Military Medical University, Chongqing, China; ^2^Department of Cardiology, People's Liberation Army General Hospital, Beijing, China; ^3^Cardiovascular Centre, Beijing Tongren Hospital, Capital Medical University, Beijing, China; ^4^The George Institute for Global Health, University of New South Wales, Sydney, NSW, Australia

**Keywords:** coronary heart disease, diabetes mellitus, social APP, WeChat, education and support intervention

## Abstract

**Background:**

Social app-assisted education and support may facilitate diabetes self-management. We aim to evaluate the effect of WeChat, a popular social app, on glycemic control in patients with coronary heart disease (CHD) and diabetes mellitus (DM).

**Methods:**

We conducted a parallel-group, open-label, randomized clinical trial that included 160 patients with both CHD and diabetes mellitus from a tertiary hospital in China. The intervention group (*n* = 80) received educational materials (information on glucose monitoring, drug usage, medication, and lifestyle) and reminders in response to individual blood glucose values *via* WeChat. The control group (*n* = 80) received usual care. The primary outcome was a change in glycated hemoglobin (HbA1C) levels over 3 months. Secondary outcomes included fasting blood glucose (FBG), systolic blood pressure, and low-density lipoprotein (LDL) cholesterol from baseline to 3 months. Analysis was conducted using a linear mixed model.

**Results:**

The intervention group had a greater reduction in HbA1C (−0.85 vs. 0.15%, between-group difference: −1.00%; 95% *CI* −1.31 to −0.69%; *p* < 0.001) compared with the control group. Change in fasting blood glucose was larger in the intervention group (−1.53 mmol/L; 95% *CI* −1.90 to −1.17; *p* < 0.001) and systolic blood pressure (−9.06 mmHg; 95% *CI* −12.38 to −5.73; *p* < 0.001), but not LDL (between-group difference, −0.08 mmol/L; 95% *CI* −0.22 to 0.05; *p* = 0.227).

**Conclusion:**

The combination of social app with education and support resulted in better glycemic control in patients with CHD and DM. These results suggest that education and support interaction *via* social app may benefit self-management in CHD and DM.

## Introduction

Diabetes mellitus (DM) currently affects more than 440 million individuals worldwide and 92.4 million adults in China, accounting for about 10.9% of Chinese adults (1). Coronary heart disease (CHD) affects ~126 million individuals and leads to 9 million deaths worldwide (2), and is the cause of ~1.7 million deaths, which is the second cause of death in China (3). The prevalence of CHD in diabetes is high and ranges from 12 to 31% among middle-aged patients with diabetes (4). It is also the major contributory cause of death, responsible for 30% of all deaths in DM (5). On the other hand, diabetes has been recognized as a risk factor for CHD, conferring up to a 4-times increasing risk of cardiovascular mortality (6). These two diseases often coexist and have a more aggressive course and worse prognosis. As China and the world enter an aging society, the burden of the two diseases increases aggressively.

The complications of cardiovascular disease and diabetes can be prevented or delayed by glycemic control. Diabetes self-management education and support (DSMES) have been proven to be efficacious and cost-effective to improve health outcomes (7). Previously, face-to-face DSMES have been shown to bring better outcomes compared with technology (telephone or online) ones (8). Recently, mobile health (mHealth) has been applied to this area and shown significant reductions in hemoglobin A1c (Hb1AC) compared with the telephone-based or usual care method (9, 10). The BlueStar mobile diabetes coach is a type 2 diabetes app that provides real-time automated educational and behavioral messages sent in response to patient-report, resulting in a mean 1.2% decline in glycated hemoglobin over 1 year (11). WeChat is the most popular communication app in China, with a penetration rate of 93% in developed cities since 2015. A meta-analysis showed that WeChat-assisted DSMES in diabetics lead to a 1.07 decline in HbA1C compared with that of controls, with reduced adverse reactions and improved satisfaction (12). However, patients with CHD and DM need to manage both diseases, which require following more lifestyle and treatment recommendations than each of the diseases alone, and evidence of social app-assisted DSMES beyond usual care in this population is still lacking. We here investigated the effects of a culturally tailored social app-assisted education and support to improve glycemic control and cardiovascular risks among patients with CHD and DM.

## Methods

### Study design

This study was a parallel-design, open-label, randomized clinical trial that evaluated a social app for assisted education and support of glucose control with a follow-up of 3 months. Individuals with both CHD and DM were recruited from the department of cardiology, Xinqiao hospitals, Chongqing, China. The trial was conducted in accordance with the Consolidated Standards Of Reporting Trials (CONSORT) checklists. All study participants have provided written informed consent. Ethical approval was obtained from the Ethics Committee of the Xinqiao Hospital Review Board (No. 2021-021-01).

### Participants

Potential participants were identified through the screening of inpatients from 2021 to 2022 with a preliminary diagnosis of CHD and DM. The inclusion criteria were ≥18 years of age, had documented diagnoses of CHD and type 2 DM, and had access to a smart mobile phone with WeChat installed and could read and send text messages through Wechat. The exclusion criteria were cognitive or communication disorders that prevented them from finishing this study.

### Randomization and intervention

Participants were randomized to either the intervention or control group in a 1:1 ratio using a random number table. Participants were aware of their treatment group. The homogenization nursing process was conducted during the hospitalization of all participants. First, patients were informed and signed the informed consent and then randomized to each group. Later blood glucose was regularly monitored 6 times per day, and treatment adjustments were made by their doctors if necessary. Moreover, relevant knowledge and health education on CHD and DM were given face-to-face before discharge. Participants in the intervention were invited to join a special WeChat account and group. They were also gotten simple training from research staff to ensure they were capable of receiving, reading, and sending relevant messages through WeChat on mobile phones.

After participants were discharged from hospital. Participants in the intervention group used the WeChat Group to receive both educational materials and reminders. This includes information about CHD and DM, glucose monitoring and control, blood pressure control, medication usage, and lifestyle recommendations. A team of cardiologists, endocrinologists, and nurses developed or shared the message. All the information was selected from relevant topic areas. Messages were drafted based on existing evidence and guidelines. The messages were sent at least 3 days per week. The control group received outpatient follow-up, as well as standard treatment. The blood glucose was recorded at 1 and 3 months in all participants. Participants were informed that they could withdraw from the study. Cardiologists and endocrinologists were also in the group chat and answered any questions that were raised. To ensure the confidentiality of all personal information, the data confidentiality policies on data collection, storage, and analysis were strictly imposed.

### Outcome measures and patient characteristics

The primary outcome was the change in glycated hemoglobin [HbA1C (hemoglobin A1C)] from baseline to 3 months. Secondary outcomes included plasma fasting blood glucose (FBG), low-density lipoprotein cholesterol (LDL-C), and systolic blood pressure (SBP) to 3 months. All blood biomarkers were measured at the Xinqiao laboratory.

### Statistical analysis

Based on previous report, we estimated that a sample size of 150 would provide 80% power to detect a 1.0% absolute difference in HbA1C change at 3 months, compared with the control group, assuming a mean HbA1C level of 8.2% at baseline (SD, 1.6%) using PASS, version 11.0 (NCSS, Kaysville, UT), for sample size calculation.

Data were collected at baseline, 1-, and 3-months follow-up visit for all participants. Epidemiological and demographic data, insurance status, and in-hospital medications were collected through the electronic medical record system. Primary and secondary outcomes, like adverse cardiovascular events such as CV death, myocardial infarction, hospitalized heart failure, and revascularization, were collected at each visit.

All analyses were conducted according to the intention to treat principle. Categorical variables were described as frequencies (percentages) and continuous variables as means ± SDs unless skewed, as medians [interquartile ranges (IQRs)]. The change of continuous variable was determined using linear mixed models. For categorical secondary outcomes, a chi-square test was used. Additionally, we performed subgroup analyses of primary outcomes by age (≤ 65 and >65 years), sex (male and female), insurance status (resident or other), hypertension status (yes and no), diabetes duration (yes and no), smoking status (current smoker or not), body mass index (BMI) (< 25 and ≥25), and insulin usage (yes and no). Results are presented as mean differences with 95% *CI*s. All tests set significance with a two-tailed α of 0.05. Statistical analyses were performed using SAS, version 9.4 (SAS Institute, Cary, NC).

## Results

**Figure 1:**
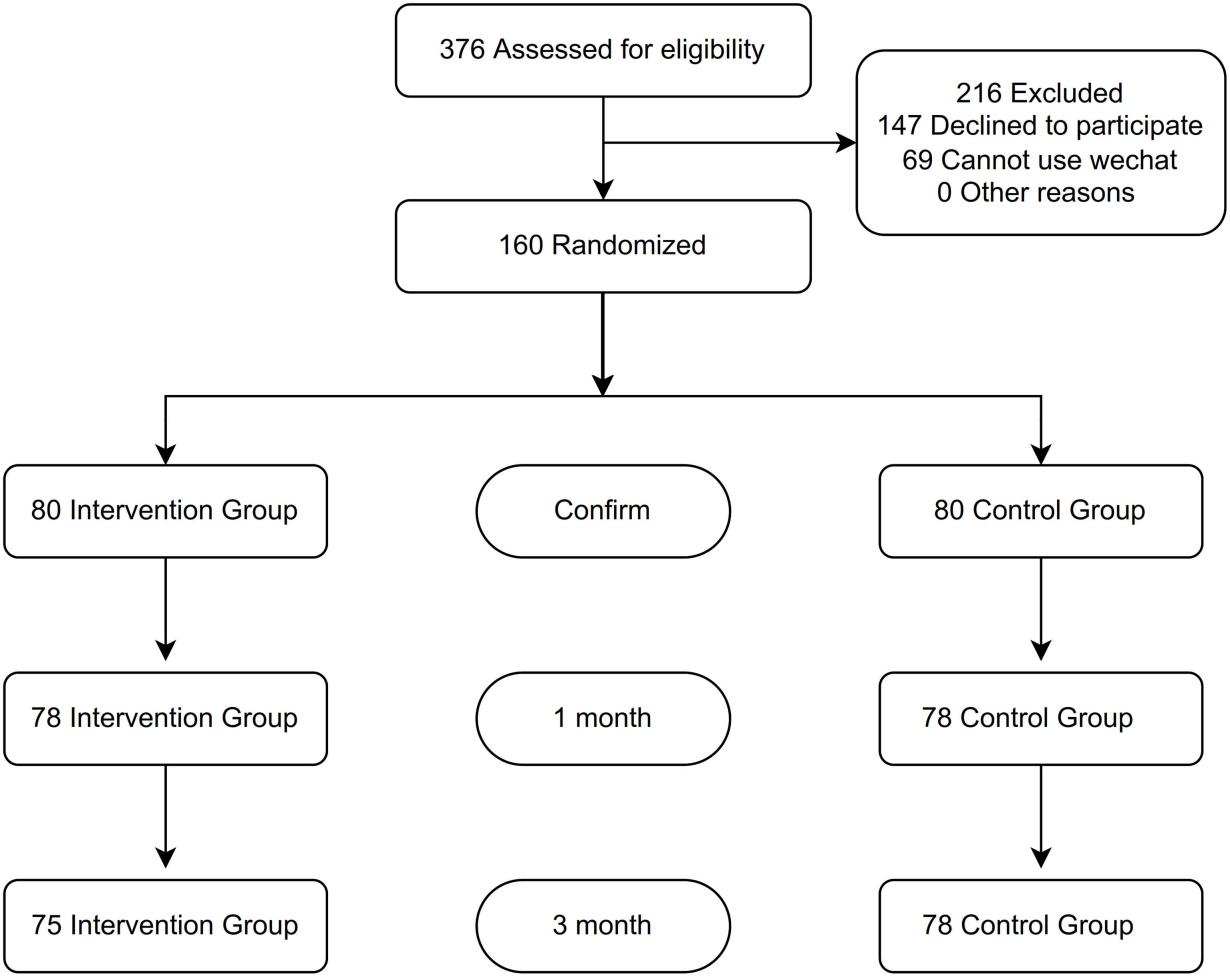
Flowchart.

Between January 2020 and December 2021, 376 patients were screened for eligibility. After excluding 147 patients who declined to participate, and 69 who could not use WeChat, a total of 160 participants were included, randomly assigned to the intervention (*n* = 80) or control group (*n* = 80; Figure 1). After randomization, 6 (3.8%) patients were lost to follow-up in total and 1 patient (0.6%) died during the study period. The median duration of follow-up was 3 months. Participants had a mean age of 65.0 years, and 34.4% were women (Table 1). The mean HbA1C level was 8.1%, mean FBG was 7.8 mmol/L, mean blood pressure was 130/75 mm Hg, mean LDL-C was 2.0 mmol/L, and mean BMI was 24.1 kg/m^2^. Baseline characteristics were similar between the intervention and control groups.

**Table 1:** Baseline participant characteristics.

	**All (*n* = 160)**	**Intervention (*n* = 80)**	**Placebo (*n* = 80)**	***P***
Age (years)	65.01 (10.05)	66.13 (10.24)	63.90 (9.80)	0.162
Female (%)	55 (34.38%)	27 (33.75%)	28 (35.00%)	0.868
High education (%)^*^	51 (31.88%)	25 (31.25%)	26 (32.50%)	0.865
Smoking (%)	62 (38.75%)	32 (40.00%)	30 (37.50%)	0.746
Resident insurance (%)	93 (58.13%)	44 (55.00%)	49 (61.25%)	0.423
Previous MI (%)	7 (4.38%)	4 (5.00%)	3 (3.75%)	0.699
Previous PCI (%)	30 (18.75%)	14 (17.50%)	16 (20.00%)	0.685
Hypertension (%)	108 (67.50%)	53 (66.25%)	55 (68.75%)	0.736
Renal disease (%)	25 (15.63%)	13 (16.25%)	12 (15.00%)	0.828
Diabetes duration (years)	8.56 (6.71)	8.09 (6.51)	9.03 (6.91)	0.379
**Blood pressure (mm Hg)**				
Systolic	129.29 (18.41)	130.13 (17.15)	128.46 (19.66)	0.570
Diastolic	75.32 (11.74)	76.48 (9.17)	74.16 (13.82)	0.214
Heart rate (bpm)	81.13 (14.40)	80.08 (15.67)	82.19 (13.02)	0.355
Body-mass index	24.13 (3.08)	24.00 (2.94)	24.26 (3.23)	0.599
LVEF (%)	58.38 (11.26)	59.27 (11.75)	57.57 (10.82)	0.377
Glycated hemoglobin (%)	8.13 (1.56)	8.25 (1.21)	8.01 (1.84)	0.327
Fasting blood glucose (mmol/l)	7.83 (2.39)	7.64 (1.93)	8.01 (2.77)	0.321
Creatinine (μmoI/L)	80.60 (65.55, 95.85)	80.45 (65.90, 99.20)	80.75 (64.75, 90.55)	0.540
LDL (mmol/L)	2.04 (0.77)	2.05 (0.65)	2.03 (0.87)	0.850
Triglycerides (mmol/L)	1.39 (0.54)	1.32 (0.43)	1.46 (0.63)	0.103
**In-hospital medication (%)**				
β-blocker (%)	124 (77.50%)	60 (75.00%)	64 (80.00%)	0.449
Oral hypoglycemic (%)	111 (69.38%)	52 (65.00%)	59 (73.75%)	0.230
Insulin (%)	69 (43.13%)	34 (42.50%)	35 (43.75%)	0.873
MI (%)	27 (16.88%)	11 (13.75%)	16 (20.00%)	0.291
Stent implantation (%)	63 (39.38%)	31 (38.75%)	32 (40.00%)	0.872

LDL, low-density lipoprotein; LVEF, left ventricular ejection fraction; MI, myocardial infarction; PCI, percutaneous coronary intervention.

* High education means junior high school or above.

### Primary outcome

**Figure 2:**
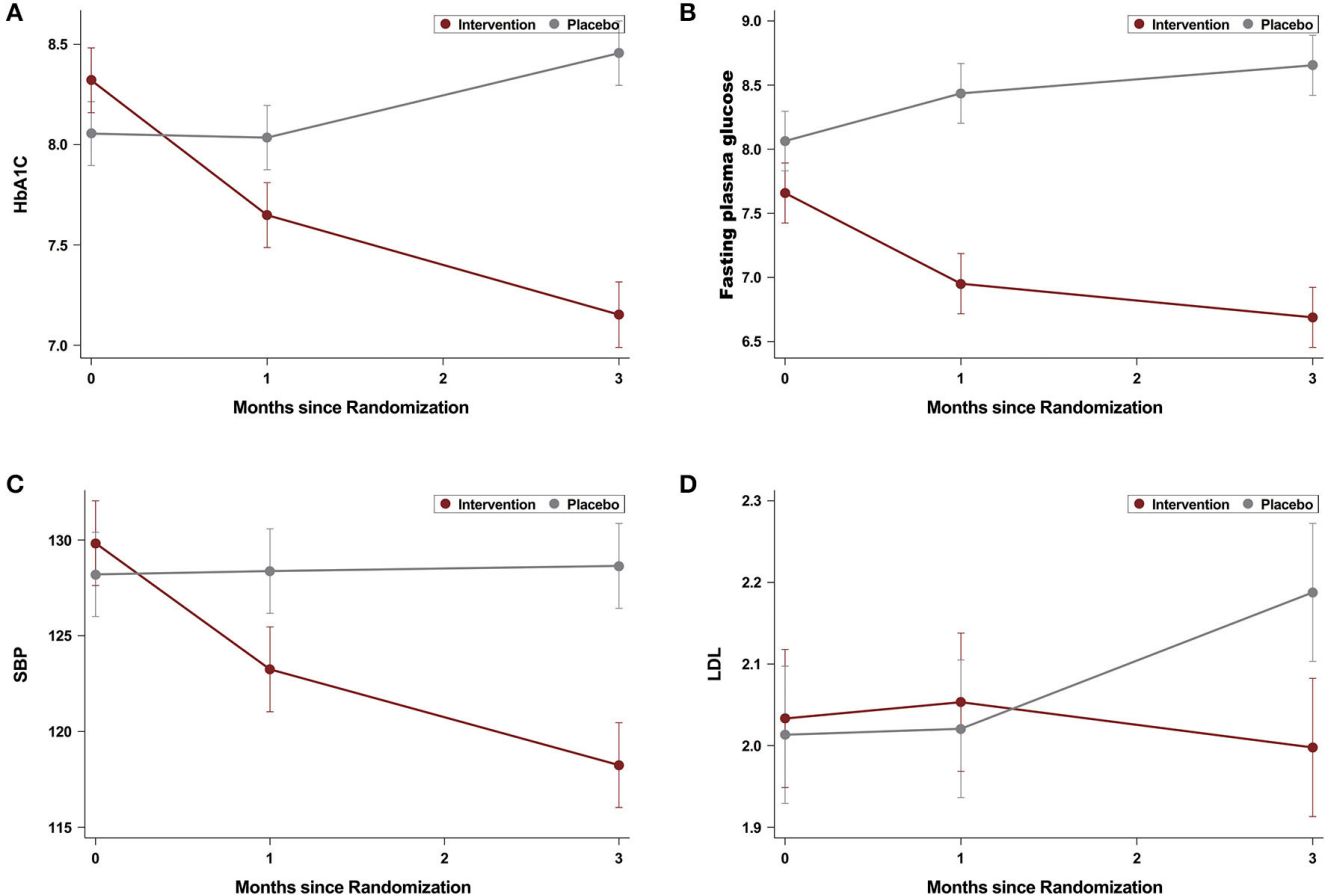
Changes in hemoglobin A1C (HbA1C) **(A)**, fasting blood glucose (FBG) **(B)**, systolic blood pressure (SBP) **(C)**, and low-density lipoprotein (LDL) **(D)** levels during 3 months of follow-up.

The main effects of the intervention on outcomes are presented in Figure 2. A significantly greater reduction in HbA1C between baseline and 3 months was observed between the intervention group and the control group, with a mean absolute difference in the HbA1C level of −1.00% (−0.85 vs. 0.15%; 95% *CI* −1.31 to −0.69%; *p* < 0.001). A significant interaction between the intervention and the subgroup levels except baseline HbA1C level ≤ 8.1 vs. >8.1% years (*p* < 0.001) were observed, a marginal interaction was observed for (left ventricular ejection fraction) LVEF (*p* = 0.027), but not for other subgroup age ≤ 65 vs. >65 years (*p* = 0.859), men vs. women (*p* = 0.362), resident vs. other (*p* = 0.372), hypertension yes vs. no (*p* = 0.072), diabetes duration < 10 vs. ≥10 years (*p* = 0.685), not smoking vs. current smoker (*p* = 0.226), BMI < 25 vs. ≥25 (*p* = 0.552), and insulin usage vs. no insulin (*p* = 0.666), respectively (Table 2). The mean difference in change from baseline to 3 months between the intervention and control groups was −0.57% (95% *CI* −0.93 to −0.20%) for participants with baseline HbA1C level ≤ 8.1 and −1.68% (95% *CI* −2.18 to −1.17%) for participants with baseline HbA1C level >8.1%. The sensitivity analysis using the analysis of covariance (ANCOVA) method got similar results, with a mean absolute difference in the HbA1C level of −0.91% (−0.80 vs. 0.11%; 95% *CI* −1.22 to −0.60%; *p* < 0.001).

**Table 2:** Subgroup analyses of the difference between the intervention and control groups in the mean change of hemoglobin A1C (HbA1C) from baseline to 3 months.

**Subgroup**	**Difference (SE)**	***P***	***P* interaction**
**Age (years)**			
≤ 65	−0.96 (0.24)	< 0.001	0.859
>65	−1.04 (0.19)	< 0.001	
**Sex**			
Female	−1.34 (0.24)	< 0.001	0.362
Male	−0.89 (0.20)	< 0.001	
**HbA**_**1C**_ **(%)**			
≤ 8.1	−0.57 (0.18)	0.002	< 0.001
>8.1	−1.68 (0.25)	< 0.001	
**Insurance**			
Resident	−1.19 (0.17)	< 0.001	0.372
Other	−0.94 (0.28)	0.001	
**Stent implantation**			
No	−1.07 (0.22)	< 0.001	0.705
Yes	−0.89 (0.24)	< 0.001	
**MI**			
No	−0.95 (0.17)	< 0.001	0.594
Yes	−1.24 (0.42)	0.008	
**Previous MI**			
No	−1.00 (0.16)	< 0.001	0.802
Yes	−1.56 (1.46)	0.364	
**Previous revasculization**			
No	−0.94 (0.18)	< 0.001	0.600
Yes	−1.30 (0.21)	< 0.001	
**Hypertension**			
No	−0.58 (0.33)	0.084	0.072
Yes	−1.24 (0.16)	< 0.001	
**Renal disease**			
No	−1.04 (0.16)	< 0.001	0.867
Yes	−1.09 (0.35)	0.005	
**Diabetes duration (years)**			
< 10	−0.94 (0.24)	< 0.001	0.685
≥10	−1.12 (0.21)	< 0.001	
**Smoking**			
No	−0.86 (0.23)	< 0.001	0.226
Yes	−1.27 (0.18)	< 0.001	
**Education**			
Low	−1.19 (0.16)	< 0.001	0.114
High	−0.63 (0.31)	0.052	
**Heart rate (bpm)**			
< 85	−0.94 (0.21)	< 0.001	0.798
≥85	−1.06 (0.23)	< 0.001	
**BMI**			
< 25	−0.96 (0.22)	< 0.001	0.552
≥25	−1.29 (0.23)	< 0.001	
**LVEF (%)**			
≤ 50	−1.18 (0.17)	< 0.001	0.027
>50	−0.55 (0.48)	0.266	
**Fasting blood glucose (mmol/l)**			
≤ 7	−0.81 (0.19)	< 0.001	0.352
>7	−1.09 (0.23)	< 0.001	
**Creatinine (μmoI/L)**			
≤ 80	−1.10 (0.21)	< 0.001	0.678
>80	−0.92 (0.23)	< 0.001	
**LDL (mmol/L)**			
≤ 2.6	−0.94 (0.19)	< 0.001	0.496
>2.6	−1.14 (0.26)	< 0.001	
**β-blocker**			
No	−1.17 (0.22)	< 0.001	0.694
Yes	−0.98 (0.19)	< 0.001	
**Oral hypoglycemic treatment**			
No	−0.74 (0.36)	0.050	0.237
Yes	−1.15 (0.17)	< 0.001	
**Insulin**			
No	−1.08 (0.15)	< 0.001	0.666
Yes	−0.93 (0.28)	0.001	

BMI, body mass index; LDL, low density lipoprotein; LVEF, left ventricular ejection fraction; MI, myocardial infarction; PCI, percutaneous coronary intervention.

### Secondary and other outcomes

Participants in the intervention group experienced a larger reduction in FBG from baseline to 3 months than the control group (−0.95 vs. 0.59 mmol/L, between-group difference, −1.53 mmol/L; 95% *CI* −1.90 to −1.17; *p* < 0.001), as well as SBP (−9.05 vs. 0.01 mmHg, between-group difference, −9.06 mmHg; 95% *CI* −12.38 to −5.73; *p* < 0.001) and DBP (−2.44 vs. 0.19, mean difference, −2.63 mmHg; 95% *CI* −4.85 to −0.42; *p* = 0.020). The LDL-C (−0.004 vs. 0.08 mmol/L, between-group difference, −0.08 mmol/L; 95% *CI* −0.22 to 0.05; *p* = 0.227) did not differ between the groups (Figure 2). A greater proportion of participants achieved HbA1C < 7% (54.7% in the intervention group vs. 16.7% in the control group; *p* < 0.001). Hypoglycemia (defined as blood glucose ≤ 3.9 mmol/L), diabetic ketoacidosis, diabetic lactate acidosis, and hyperosmolar non-ketotic diabetic syndrome were not observed in all the participants. During a 3-months follow-up period, a marginal difference was observed in the adverse cardiovascular event (0 in the intervention group and 6 for the placebo group, *p* = 0.028, Table 3).

**Table 3:** Clinical outcome at 3 months after initial treatment.

	**Intervention**	**Placebo**	***p* value**
Total event	0	6	0.0284
CV death	0	1	
Angina	0	2	
MI	0	1	
Hospitalized HF	0	1	
Revascularization	0	1	

CV, cardiovascular; HF, heart failure; MI, myocardial infarction.

## Discussion

Adequate glycemic control of diabetes is still lacking. The HbA1C target < 7% is only achieved in about 30–50% of patients with diabetes (13, 14). The situation may be even worse in patients with both CHD and diabetes as they need to pay more attention to CHD as symptoms are more apparent rather than diabetes. From this analysis, we found that a social app-assisted education and support improved glycated hemoglobin by −1.0% over 3 months. The magnitude of improvement observed in this study is consistent with the average reduction 1.07% in a recent meta-analysis, which reported glucose level after self-management among patients with type 2 diabetes *via* the WeChat application (12). Our result showed a larger reduction in population with poorly controlled glycated hemoglobin (>8.1%) than in people with less severe ones ( ≤ 8.1%). Fasting plasma glucose and blood pressure were also improved, but not LDL-C, and more adverse CV events were observed in the control group than in the treatment group.

Smart mobile phones are popular, according to Deloitte Global mobile consumer trends, more than 80% of the population in the world has a smartphone, and in China, this is higher (about 86%) (15). WeChat is the only instant messaging app in China to have over one billion active users, having similar functions to Whatsapp and Facebook messenger. In our study, social app-assisted education and support could improve blood glucose control more than the control group. Although social app itself could not benefit blood glucose control, it helps patients and medical staff to establish a connection out of hospital. The improvement could be explained with the following reasons. First patients could receive relevant education and support *via* not only texts and voices, but also pictures and videos. The addition facilitates patients' understanding of these materials (16). Second, the group chat function support one medical staff share material and corresponds to multiple patients, and patients can discuss with each other. Third, this method could let patients communicate with their doctors in a real-time manner. This prevents patients from making the wrong choice when instant support is needed. Fourth, this is a simple, low-cost approach to augment existing public health services compared with other methods, such as adding wearable devices. Therefore, the combination of usual care and social app-assisted education and support would be more conducive to patient self-management.

The social app-assisted education and support also has some disadvantages. First, patients should have a smart phone and connected to the network, otherwise the information could not be obtained. Second, this would add extra work to already busy medical staff, limiting its use.

The between-group difference is a combination of the decrease in HbA1C in the intervention group and mild increase in the control group. Similar trend are shown for fasting plasma glucose and blood pressure but not LDL. The absence of LDL may be due to patients who could not measure their cholesterol frequently by themselves at home, and therefore do not pay enough attention.

The results showed that social app-assisted education and support could improve glycemic control in diabetic patients with CHD, similar to those without CHD. Moreover, expansion of such education and support to CHD but not only diabetes could lead to improved blood pressure control and the potential to reduce adverse cardiovascular events. In fact, smartphone and social media-based cardiac rehabilitation and secondary prevention in CHD could improve 6-min walk distance (17), help quitting drinking and smoking (18), improve medication adherence, and blood pressure control (19). Thus social app-assisted education and support could benefit two diseases at a single shot in such situation.

Our study has several limitations. First, although our intervention provided lifestyle guidance regarding food, exercises, emotions, and other risk management behaviors, there was no specific measurement indicator about the effectiveness on lifestyle changes, such as resting energy expenditure, physical activity levels, and dietary intake. Additional research is needed to answer such questions. Second, our primary outcomes are not hard endpoint, such as recurrent myocardial infarction or CV death. Third, we only follow participants for a time of 3 months, whether longer intervention could translate into larger benefits is still unknown. Fourth, self-reported measures are subjected to recall biases. Last, our sample size was relatively small and we only included Chinese participants. Future large samples and studies from other countries are needed to confirm the reliability of the results.

## Data availability statement

The raw data supporting the conclusions of this article will be made available by the authors, without undue reservation.

## Ethics statement

The studies involving human participants were reviewed and approved by Ethical approval was obtained from Ethics Committee of Xinqiao Hospital Review Board (No. 2021-021-01). The patients/participants provided their written informed consent to participate in this study.

## Author contributions

JZ and J-WL conceived the concept of the study and drafted the manuscript. DHQ and J-WL performed the statistical analyses and drafted the manuscript. HMZ and YZL collected baseline and follow-up data. All authors contributed to the article and approved the submitted version.

## Funding

This study was supported by the Chongqing overseas students returning home entrepreneurship and innovation support plan (41422145).

## Conflict of interest

The authors declare that the research was conducted in the absence of any commercial or financial relationships that could be construed as a potential conflict of interest. All authors were employed by PATH.

## Publisher's note

All claims expressed in this article are solely those of the authors and do not necessarily represent those of their affiliated organizations, or those of the publisher, the editors and the reviewers. Any product that may be evaluated in this article, or claim that may be made by its manufacturer, is not guaranteed or endorsed by the publisher.
